# Incidence and clinicopathologic features of gastrointestinal stromal tumors. A population-based study

**DOI:** 10.1186/1471-2407-7-230

**Published:** 2007-12-20

**Authors:** Claudia Mucciarini, Giulio Rossi, Federica Bertolini, Riccardo Valli, Claudia Cirilli, Ivan Rashid, Luigi Marcheselli, Gabriele Luppi, Massimo Federico

**Affiliations:** 1 Department of Oncology and Haematology, University of Modena and Reggio Emilia, Modena, Italy; 2 Section of Pathologic Anatomy, University of Modena and Reggio Emilia, Modena, Italy

## Abstract

**Background:**

Although the diagnostic criteria and pathogenesis of gastrointestinal stromal tumors (GIST) have recently been elucidated, knowledge of the epidemiology of this malignancy is still limited. This study examined the incidence of GIST in the province of Modena, including pathologic features and clinical outcome.

**Methods:**

Gastrointestinal mesenchymal tumors identified by the Modena Cancer Registry between 1991 and 2004 were analyzed with an immunohistochemical panel that included staining for CD-117 and PDGFRα. Size, mitotic rate, and other pathologic parameters were recorded. Each tumor was categorized into National Institutes of Health risk categories (very low, low, intermediate, and high risk).

**Results:**

One hundred twenty-four cases were classified as GIST. The age-adjusted incidence rate was 6.6 per million. Seventy-five percent of patients were symptomatic; 34% had a previous or concomitant history of cancer. High-risk features were present in 47% of cases. Seventy-eight percent were submitted to radical surgery. After complete resection, the 5-year disease-free survival rates were 94%, 92%, 100%, and 40% for patients at very low, low, intermediate, and high risk, respectively. In multivariate analysis, high risk was the main predictor of recurrence.

**Conclusion:**

This population-based study shows that the incidence of GIST in Northern Italy is comparable to that reported in other European countries. Survival was favorable in lower risk categories and in most of the resected cases. In our study, resected patients at very low, low, and intermediate risk had a similar outcome. Our data support the need to consider high-risk patients after complete surgical resection for treatment with the best available approach.

## Background

The term stromal tumor was first introduced by Mazur and Clark [1] in 1983 to define a group of gastric mesenchymal tumors that were not clearly differentiated by immunohistochemistry and ultrastructure and that were previously thought to be derived from smooth muscle of the gastrointestinal wall. In 1998, Kindblom *et al.*[2] and Hirota *et al.*[3] independently determined that these neoplasms are significantly immunoreactive for CD117, a polyclonal antibody recognizing the type III tyrosine kinase KIT, which is encoded by the proto-oncogene *c-kit*. Since in the normal gastrointestinal tract KIT is exclusively expressed by the interstitial cells of Cajal (ICC), specialized cells that interact with neural and muscular structures that have a pacemaker activity, the authors suggested that gastrointestinal stromal tumors (GISTs) might derive from ICC or from stem cells differentiating toward an ICC phenotype [2]. Moreover, Hirota *et al.*[3] demonstrated gain-of-function mutations involving KIT that result in ligand-independent KIT receptor activation.

GISTs are the most common gastrointestinal soft tissue malignancies. They mainly occur in the stomach (60–70%) and small intestine (20–30%); esophageal, colon, and rectal GISTs are relatively infrequent. Exceptionally, GISTs occur in the omentum, mesentery, and retroperitoneum [4]. The median age of patients with GIST is 60–69 years, and symptoms mainly relate to tumor size and site. The epidemiology of GISTs reflects increased interest in the tumors and improved ability to perform exact diagnosis. Given that an exact definition of GIST was developed only quite recently and many cases included in the largest series are from consultation files, few population-based studies have highlighted the real incidence and clinicopathologic characteristics of GISTs.

The aim of this work was to better define the epidemiology and clinicopathologic features of GISTs by examining all cases of soft-tissue tumors originating from the gastrointestinal tract that were diagnosed in the province of Modena (Italy) between 1991 and 2004. We reviewed these cases for pathologic features, risk stratification, and clinical outcome.

## Methods

We considered all cases of gastrointestinal soft tissue and/or spindle cell tumor diagnosed in residents of the province of Modena (population 633,993 at 2001 census) between 1991 and 2004. Cases were identified using the Modena Cancer Registry database and the archival files of the Section of Pathologic Anatomy of the University of Modena and Reggio Emilia.

Two pathologists (GR and RV) used a multi-headed microscope to review all the hematoxylin-eosin stained slides available for each tumor (mean, 5.5; range, 1–15). In addition, several 4-μm-thick sections obtained from a representative formalin-fixed and paraffin-embedded block of each tumor were cut for immunohistochemical analysis.

Immunohistochemistry was performed using an automated immunostainer (Benchmark, Ventana, Tucson, AZ). All cases were tested with the following panel of antibodies: CD117 (polyclonal, A4502; Dako, Glostrup, Denmark; 1:200 dilution; no antigen retrieval), PDGFRα (polyclonal, sc-338; Santa Cruz Biotechnology, Santa Cruz, CA; 1:150 dilution; microwave treatment), smooth-muscle actin (monoclonal, 1A4; Biogenex, San Ramon, CA; 1:20; no antigen retrieval), desmin (monoclonal, D33; Dako; 1:10; microwave treatment), S100 (polyclonal, RB044-A; NeoMarkers, Fremont, CA; 1:5; no antigen retrieval), and CD34 (monoclonal, QB-END/10; Novocastra, Newcastle upon Tyne, UK; 1:40; microwave treatment) and cytokeratins (monoclonal, MNF116; Dako; 1:100; protease treatment).

Diagnosis of GIST was established based on clear immunostaining for CD117 or PDGFRα in an adequate clinical and morphologic context, as previously described [5]. All cases had sufficient tumor material for immunohistochemical analysis using the above-mentioned panel of antibodies. All cases were also studied by direct sequencing PCR of the *c-kit *exons 9, 11, 13, 17 as well as PDGFRα exons 12 and 18, as previously described [6]. Briefly, 89 cases showed somatic mutations on *c-kit *exon 11, 36 on exon 9, 4 on exon 13 and none at exon 17. PDGFRα mutations were detected in 2 cases (both on exon 12) and 6 cases were "wild-type".

Apart from GIST, we originally reviewed also other 67 neoplasms somehow mimicking GIST, then entering into its differential diagnosis, as follows: 27 smooth muscle tumors (18 leiomyomas, 9 leiomyosarcomas); 23 desmoids; 5 spindle cell sarcomatoid carcinomas; 5 inflammatory fibroid polyps (so-called Vanek's tumor); 3 schwannomas; 2 solitary fibrous tumors and 2 inflammatory pseudotumors.

Differential diagnosis was performed by light-microscope morphologic analysis, immunostaining using the above panel and in selected cases by means of mutational analysis.

The tumor risk category was defined by assessing tumor size and mitotic count, as suggested by the consensus guidelines of the National Institutes of Health-(NIH-NCI) workshop[7]. Clinical information was retrieved from hospital records. Follow-up was performed by active search of all discharge letters, review of hospital records, and interview of general practitioners. Information on patients' vital status was obtained from official population registries.

Survival was calculated from the day of diagnosis until death or the last time the official population registries were consulted (June 2006). Disease-free survival was calculated from the date of radical surgery until tumor recurrence. Overall survival and disease-free survival were calculated using the Kaplan-Meier method and analyzed using the log-rank test. Relative survival was determined according to the Hakulinen method [8].

Univariate and multivariate Cox proportional hazard regression analyses were used to analyze factors that may predict tumor recurrence. All calculations were performed with the Stata 8 package. Differences were considered significant at *P*-values < 0.05. Patients with metastatic disease at the time of diagnosis were grouped into the category "overtly malignant". Analysis of the risk of recurrence was performed separately for all patients (including those with overtly malignant disease) using Fisher's exact test; multivariate Cox proportional hazard regression analyses were used to analyze the risk of recurrence for patients that underwent surgical resection. A cumulative and smoothed risk of metastases after surgery was calculated. Receiver operating characteristics methodology [9] has been used to evaluate the ability of Fletcher's score in individuate risk of recurrence after resection, overall metastases, and death.

All information were collected and used to perform anonymous and aggregate statistical analysis. According to the Italian Laws, approval by a formal ethics committee is not required for this type of study.

## Results

Diagnosis of GIST was established in 137 cases, 124 of whom were resident in the province of Modena. Only these 124 patients were included in the analysis. Sixty-six of these patients (53.2%) were men, and 58 (46.8%) were women. The median age at presentation was 69 years (range: 30–90 years). The most common primary site was the stomach (63% of all GISTs), followed by the small intestine, retroperitoneum, colon-rectum, esophagus, and anus (Table 1). Overall, GISTs represented 0.2% of all invasive cancers for both genders. Moreover, they accounted for 1.8% of all stomach cancers and 13% of all cancers arising in the small intestine. The crude incidence rate was 1.42 per 100,000, 1.55 per 100,000 for men, and 1.30 per 100,000 for women. The age-standardized rate (ASR) according to the world standard population was 0.66 per 100,000 (men: 0.84 per 100,000, women: 0.49 per 100,000).

**Table 1:** Summary of results

	**Number**	**%**
Gender	124	100
Male	58	46.7
Female	66	53.2

Age	124	100
≤ 60 years	32	25.8
61–70 years	38	30.6
> 70 years	54	43.5

Anatomical distribution	124	100
Esophagus	2	1.6
Stomach	78	62,9
Small intestine	29	23.4
Colon	4	3.2
Rectum	2	1.6
Anus	1	0.8
Retroperitoneum	8	6.5

Clinical symptom	93	75
Intestinal bleeding	31	25
Abdominal pain	44	35.5
Dyspepsia	20	16.1
Nausea/vomiting	15	12.1
Constipation/diarrhea	11	8.9
Abdominal mass	10	8.1
Worsening general condition	30	24.2
Urologic dysfunction	1	0.8
Iron deficiency anemia	19	15.3

One hundred ten tumors were KIT positive (88.7%) while 14 GISTs were KIT negative. In these tumors, the neoplastic cells immunoreacted for PDGFRα and in 2 a PDGFRα mutation was found. We also noted mutually exclusive expression and mutational set-up for *c-kit *and PDGFRα.

The most frequent symptoms were abdominal pain (36%), gastrointestinal bleeding (25%), worsening of general condition (24%), and dyspepsia (16%) (Table 1). Thirty-one patients (25%) were asymptomatic. In 39 patients (31.5%), GIST was incidentally discovered during endoscopy or laparotomy performed for other reasons such as colon polyposis or synchronous gastrointestinal cancer. Forty-two patients (33.9%) had a previous history of cancer.

Ninety-seven patients (78.2%) underwent radical surgery, while 27 patients (21.8%) were unresectable due to diffuse metastatic disease at diagnosis. Eighty-three GISTs (66.9%) were of spindle-cell morphology, 19 cases (15.3%) were epithelioid, and 17 cases (13.7%) were of mixed histotype. In the remaining cases (4%), the small bioptic specimen did not permit exact assessment of histopathologic features.

In resected tumors, the mitotic count was less than 5 per 50 high-power fields (HPF) in 53 patients (54.6%), between 6 and 10 per 50 HPF in 17 patients (17.5%), and more than 10 per 50 HPF in 24 patients (24.7%). Tumor size was distributed as follows: 17 cases (17.5%) were less than 2 cm, 21 cases (21.6%) were between 2 and 5 cm, 27 cases (27.8%) were between 5 and 10 cm, and 32 cases (33%) were greater than 10 cm.

The prevalence of patients at very low, low, intermediate, and high risk according to Fletcher's score were 15.3%, 15.3%, 17.7%, and 51.6%, respectively. Overtly malignant tumors accounted for 29 of 124 GISTs (23%), and represented 5%, 16%, 23%, and 31% of the cases in the very low, low, intermediate, and high risk groups, respectively.

**Figure 1:**
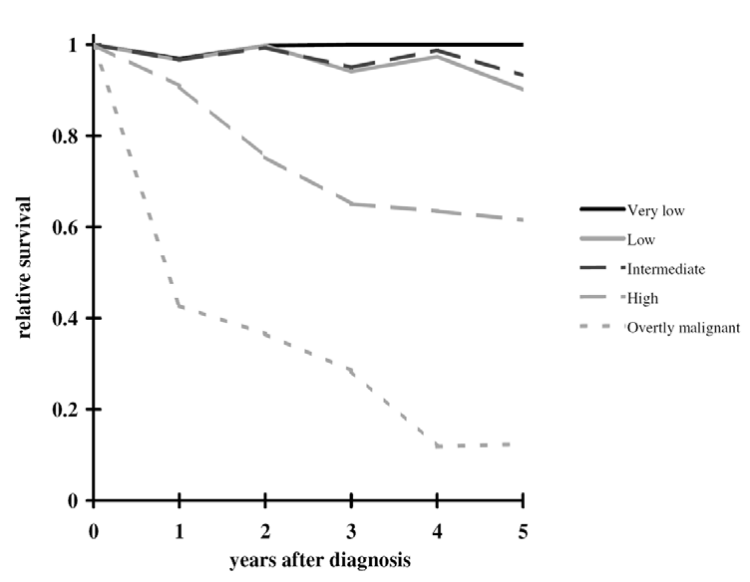
Relative survival for all patients (N = 124) according to very low (N = 18), low (N = 16), intermediate (N = 17), higk risk (N = 44) and overtly malignant (N = 27) categories.

The median follow-up for living patients was 63 months. The overall 5- and 10-year relative survival rates were 65.8% (95% CI: 53.9–76.4%) and 60.4% (95% CI: 43.3–77.1%), respectively. The 5-year relative survival rates were 100% (95% CI: 77.9–115%), 90.1% (95% CI: 50.4–108.2%), 93.2% (95% CI: 54.4–110.3%), and 61.5% (95% CI: 41.3–78.6%) for patients in the very low, low, intermediate, and high risk categories, respectively, and 12.4% (95% CI: 2.3–32.5%) for overtly malignant GISTs (Figure 1).

**Figure 2:**
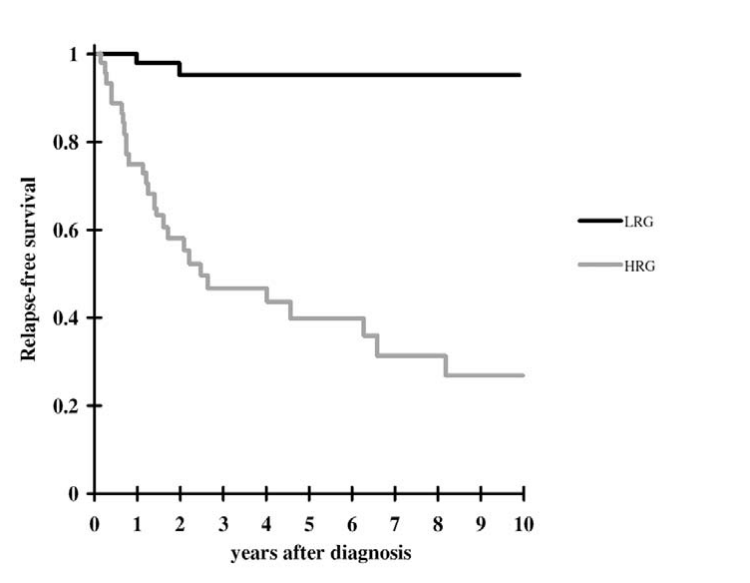
Ten-year relapse-free survival (RFS) for 97 patients with complete resection of primary tumor according to very low (N = 18), low (N = 16), intermediate (N = 17) and higk (N = 46) risk categories.

The 5-year relative survival rates were 84.6% (95% CI: 72–93.2) for patients treated with radical surgery (N = 97) and 13.8% (95% CI: 0–30.7%) for those without radical surgery (N = 27). Table 2 shows the distribution of tumor recurrence after intervention in the group of 97 patients treated with complete resection of the primary tumor. Given the superimposable relapse rate of patients falling into the very low, low, and intermediate risk groups, all three risk categories were grouped together into a unique lower-risk group (LRG), whereas the high-risk group (HRG) corresponds to the original NIH category (receiver operating characteristics area: 82.6%). The relapse-free survival rate was 95% for LRG patients and 40% for HRG patients, respectively (*P *< 0.001, Figure 2).

**Table 2:** Relapse-free survival (RFS) in 97 patients with complete resection of the primary tumor

	**Number **	**5-year DFS (%)**	*P*-value*
Age			
≤ 60 years	26	66	0.953
61–70 years	33	72	
> 70 years	38	67	
Tumor location			
Stomach/small intestine	72	73	0.002
Other	25	36	
Mitotic count			
< 5 per 50 HPF	56	89	< 0.001
5–10 per 50 HPF	24	80	
> 10 per 50 HPF	17	16	
Size			
< 2 cm	17	100	< 0.001
2–5 cm	21	90	
5–10 cm	27	67	
> 10 cm	32	39	
Gender			
Male	53	75	0.420
Female	44	63	
Symptoms			
Yes	33	73	0.348
No	64	52	
Tumor risk category			
Very low	18	94	< 0.001
Low	16	94	
Intermediate	17	100	
High	46	40	
Condensed risk group^#^			
LRG	51	95	< 0.001
HRG	46	40	

*Fisher's exact test

^#^LRG, very low, low, and intermediate groups; HRG, high risk group

All metastatic events were evaluated with respect to age, location, mitoses, size, gender, symptoms, and Fletcher's score by grouping together overtly malignant tumors and tumors that recurred after resection. In univariate analysis, location other than the stomach and small intestine, mitotic count higher than 5 per 50 HPF, and tumor size greater than 10 cm were associated with increased overall risk of recurrence. NIH and condensed risk categories also significantly correlated with recurrence (Table 3). The multivariate Cox regression model confirmed the independent prognostic role of tumor risk category (OR: 12.3, 95% CI: 5.2–29) and, alternatively, both mitotic count higher than 5 per 50 HPF (OR: 6.2, 95% CI: 2.3–17.2) and tumor size greater than 10 cm (OR: 3.3, 95% CI: 1.4–7.6).

**Table 3:** Distribution of metastatic events according to tumor and patient characteristics

	**Number of events/number of cases**	**%**	*P*-value*
Age			
≤ 60 years	14/32	44	0.825
61–70 years	17/38	45	
> 70 years	27/54	50	
Location			
Stomach	32/78	41	0.029
Small intestine	13/29	45	
Other	13/17	76	
Mitotic rate			
< 5 per 50 HPF	12/62	19	< 0.001
5–10 per 50 HPF	16/27	59	
> 10 per 50 HPF	30/35	86	
Size			
< 2 cm	3/20	15	< 0.001
2–5 cm	8/27	30	
5–10 cm	15/35	43	
> 10 cm	32/42	76	
Gender			
Male	26/58	45	0.721
Female	32/66	48	
Symptoms			
Yes	45/93	48	0.678
No	13/31	42	
Tumor risk category			
Very low	2/19	10	< 0.001
Low	4/19	21	
Intermediate	5/22	23	
High	47/64	73	
Condensed risk group^#^			
LRG	11/60	18	< 0.001
HRG	47/64	73	

*Log-rank test

^# ^LRG, very low, low, and intermediate groups; HRG, high risk group

## Discussion

This is the first population-based epidemiological study evaluating the features of malignant GIST in Italy. Table 4 shows a summary comparison between our results and those published in other similar series. Our findings, based on the Modena Cancer Registry data, indicate an annual crude incidence rate in the province of Modena of 14.2 new cases per million people. Nilsson *et al.*[10] estimated the rate at 14.5 per million. Tran *et al.*[11] estimated the 2000 U.S. census ASR at 6.8 per million, much lower than that estimated for the province of Modena (after age adjustment, according to the 2000 U.S. standard population, the Modena ASR was 9.8 per million, 11.9 per million in men, and 8.0 per million in women). Recently, Tryggvason *et al.*[12] investigated all cases of malignant GIST diagnosed from 1990 to 2003 in Iceland (300,000 inhabitants) and estimated an overall world ASR of 1.1/100000. Even more recently, Rubio *et al.*[13] reported a population-based study in Girona, Spain (553 000 inhabitants) that demonstrated an overall world ASR of 0.65/100000 from 1994 to 2001. These comparisons show an incidence similar to that reported in Spain and Sweden. The difference between Modena and Iceland may relate to different ability in identifying lower-grade lesions, which may affect the incidence in the very low-risk and low-risk categories. Finally, the difference between our results and those estimated in the U.S. [11] may be due to some previously observed limitations of the cited U.S. study.

**Table 4:** Incidence and outcome of GISTs from different population-based studies

	**Italy**	**Sweden **[10]	**USA **[11]	**Iceland **[12]	**Spain **[13]
Covered population					
Area	Modena	Western Sweden	12 SEER registries	Iceland	Girona
Population	633,993	1.3–1.6 million	38.7 million	300,000	553,661
Period	1991–2004	1983–2000	1992–2000	1990–2003	1994–2001
Incidence					
Number of cases	124	288	1,458	114	46
Crude rate^#^	1.42	1.45	n.a.	n.a.	n.a.
World ASR^#^	0.66	n.a.	n.a.	1.1	0.65
US 2000 ASR^#^	0.98	n.a.	0.68	n.a.	n.a.
5-years relative survival (%)					
All cases	65.8	n.a.	45	n.a.	74.7
Very low risk	100	n.a.	n.a.	n.a.	94.9^(*a*)^
Low risk	90.1	n.a.	n.a.	n.a.	
Intermediate risk	93.1	n.a.	n.a.	n.a.	100
High risk	61.5^(*b*)^	n.a.	n.a.	n.a.	21.4
Overly malignant	12.4	n.a.	n.a.	n.a.	n.a.

^#^per 100,000 inhabitants

^(a)^Very low risk + low risk groups

^(b)^Excluding overly malignant diseases

The significant immunoreactivity of GIST to KIT (CD117) has permitted a new nosologic definition. KIT immunostaining is an important method for diagnostic distinction of GIST from leiomyoma, leiomyosarcoma, and schwannomas, which typically do not exhibit KIT positivity [14]. In our series, most cases were KIT positive. However, the KIT-negative cases were immunoreactive for PDGFRα, providing immunohistochemical confirmation that KIT and PDGFRα exhibit mutually exclusive expression, and not only mutually exclusive mutations [15]. The frequency of PDGFRα mutations in our series is slightly higher than that in other series [4] and suggests that the assessment of both KIT and PDGFRα mutations can be helpful in establishing a more correct diagnosis.

The clinical presentation of GISTs varies according to the site and size of neoplasm. In our study, GISTs were mostly located in the stomach and small intestine. The anatomic distribution seemed unrelated to patient outcome. The impact of anatomic location of GIST on survival is controversial. In fact, in some studies, GISTs arising in the stomach were less aggressive than GISTs arising in the lower gastrointestinal tract [16], while other studies found no differences in outcome[17].

In our series, as also reported in the Swedish study [10], the majority of large GISTs were detected after the onset of symptoms, while small GISTs were detected incidentally during surgery performed for other reasons. Our results confirm that the most common symptoms (in descending order of frequency) are abdominal pain, intestinal bleeding, and dyspepsia [18].

Tumor size significantly correlated with mitotic count; larger tumors usually had higher mitotic counts and were frequently unresectable. As a result, GIST cases with tumor size > 10 cm have poorer survival [19]. These two histologic criteria are important to define the biological behavior of a tumor and risk category for prognosis. Conventional histologic factors do not predict the malignant potential and malignant actions of GISTs. Recently, the descriptors of the malignant behavior of GISTs, benign and malignant, were substituted with low, intermediate, and high risk. Our results confirm that prognosis is significantly better for patients in very low-, low-, and intermediate-risk categories than for patients in high-risk categories.

Complete tumor resection is another important factor related to survival [20,21] In this study, for GISTs with metastasis or with incomplete resection, survival was poor after surgery alone. After radical surgery, the majority of relapses occurred in patients with tumors classified as high risk. It seems to be no differences between the three lower risk categories when tumor is resectable while limited differences can be found when considering metastatic events in all patients. These distinction and obviously limitations due to the small number of patients should all be considered when comparing outcome of intermediate risk category in this study with other series. However, Cox multivariate analysis demonstrated the clinical usefulness of both the NIH-NCI and condensed (LRG and HRG) risk categories. Our data also support an alternative simplified risk score based on the cumulative contribution of two prognostic factors: mitotic count higher than 5 per 50 HPF and tumor size greater than 10 cm.

## Conclusion

Early diagnosis and complete resection remain the standard of care, but high-risk patients must be considered for enrollment in clinical trials of adjuvant treatment. Our population-based study suggests that the incidence of GIST in Italy is comparable to that reported in Spain and other European countries. However, it will be useful to perform more population-based studies to obtain more details about epidemiology and to enable prediction of the future incidence of these neoplasms.

## Competing interests

The author(s) declare that they have no competing interests.

## Authors' contributions

CM, CC, and FB collected data for this study; CM also performed part of the descriptive analysis. IR performed epidemiological analysis as well as the survival analysis. LM and CC reviewed the multivariate analysis. GR and RV reviewed all tumor slides. GL critically revised the draft. FM participated in the study design and helped draft the manuscript. All authors read and approved the final manuscript.

## Pre-publication history

The pre-publication history for this paper can be accessed here:


